# Microsensor measurements of hydrogen gas dynamics in cyanobacterial microbial mats

**DOI:** 10.3389/fmicb.2015.00726

**Published:** 2015-07-21

**Authors:** Michael Nielsen, Niels P. Revsbech, Michael Kühl

**Affiliations:** ^1^Section of Microbiology, Department of Bioscience, Aarhus UniversityAarhus, Denmark; ^2^Marine Biological Section, Department of Biology, University of CopenhagenHelsingør, Denmark; ^3^Plant Functional Biology and Climate Change Cluster, University of Technology, Sydney, UltimoNSW, Australia

**Keywords:** hydrogen, oxygen, sulfide, pH, irradiance, microsensor, microbial mat, cyanobacteria

## Abstract

We used a novel amperometric microsensor for measuring hydrogen gas production and consumption at high spatio-temporal resolution in cyanobacterial biofilms and mats dominated by non-heterocystous filamentous cyanobacteria (*Microcoleus chtonoplastes and Oscillatoria sp.*). The new microsensor is based on the use of an organic electrolyte and a stable internal reference system and can be equipped with a chemical sulfide trap in the measuring tip; it exhibits very stable and sulfide-insensitive measuring signals and a high sensitivity (1.5–5 pA per μmol L^-1^ H_2_). Hydrogen gas measurements were done in combination with microsensor measurements of scalar irradiance, O_2_, pH, and H_2_S and showed a pronounced H_2_ accumulation (of up to 8–10% H_2_ saturation) within the upper mm of cyanobacterial mats after onset of darkness and O_2_ depletion. The peak concentration of H_2_ increased with the irradiance level prior to darkening. After an initial build-up over the first 1–2 h in darkness, H_2_ was depleted over several hours due to eﬄux to the overlaying water, and due to biogeochemical processes in the uppermost oxic layers and the anoxic layers of the mats. Depletion could be prevented by addition of molybdate pointing to sulfate reduction as a major sink for H_2_. Immediately after onset of illumination, a short burst of presumably photo-produced H_2_ due to direct biophotolysis was observed in the illuminated but anoxic mat layers. As soon as O_2_ from photosynthesis started to accumulate, the H_2_ was consumed rapidly and production ceased. Our data give detailed insights into the microscale distribution and dynamics of H_2_ in cyanobacterial biofilms and mats, and further support that cyanobacterial H_2_ production can play a significant role in fueling anaerobic processes like e.g., sulfate reduction or anoxygenic photosynthesis in microbial mats.

## Introduction

Molecular hydrogen (H_2_) is produced during fermentative anaerobic degradation of organic matter (Conrad, 1988). Formation of H_2_ as a by-product of nitrogenase activity is also described as a major H_2_ producing process (Bothe et al., 2010). In excess of energy and reducing power, photosynthetic bacteria can also photo-produce H_2_ (Gest and Kamen, 1949; Warthmann et al., 1992). Hydrogen is a very good energy source that is readily reacting with O_2_ (chemically or catalyzed by “Knallgas”-bacteria) or is consumed by anaerobic mineralization processes, e.g., as an electron donor in sulfate reduction and in methanogenesis (Hoehler et al., 2002). Anoxygenic photosynthetic bacteria can also utilize hydrogen as an electron donor (Overmann and Garcia-Pichel, 2000).

Efficient inter-species hydrogen transfer in consortia of microorganisms allows for syntrophic processes, which separately would otherwise be energetically unfavorable (Wolin, 1982; Hoehler et al., 2001). Consequently, only very low H_2_ concentrations are detected in most natural environments. Higher H_2_ levels can, however, be found in special environments like the digestive tracts of termites (Ebert and Brune, 1997) or in legume nodules harboring N_2_-fixing bacteria (Witty, 1991). Geothermal features can also exhibit high levels of H_2_, and hydrogenotrophs are widespread in many hot springs, where H_2_ metabolism can be predominant (Spear et al., 2005).

Hydrogen production in cyanobacteria has been known for a long time (Jackson and Ellms, 1896; Benemann and Weare, 1974; Oschchepkov et al., 1974) and has been studied for a large number of strains and in different environments (Lambert and Smith, 1981; Houchins, 1984; Kothari et al., 2012; Otaki et al., 2012). In recent years, cyanobacterial H_2_ production has also become a major research topic in connection with the search for new clean energy generating processes (e.g., Lee et al., 2010; Hallenbeck, 2012). Production of H_2_ in cyanobacteria is primarily associated with N_2_ fixation, where H_2_ is a major by-product (Bothe et al., 2010), or due to dark fermentation of storage products accumulated during daytime photosynthesis (Moezelaar et al., 1996; Stal and Moezelaar, 1997). In a survey of different cyanobacterial strains, Kothari et al. (2014) demonstrated that fermentative pathways and bidirectional NADH-linked [Ni-Fe] hydrogenases are of prime importance for H_2_ production under dark anoxic conditions in the filamentous non-heterocystous cyanobacteria *Microcoleus chtonoplastes* and *Lyngbya aestuarii* that form dense microbial mats in coastal and hypersaline environments; these species exhibited higher production rates and reached much higher steady state H_2_ concentrations than many other cyanobacteria. Bidirectional hydrogenases are also involved in light-driven H_2_ formation under anoxia via direct biophotolysis, i.e., the light-driven splitting of water (Appel et al., 2000).

Earlier reports of significant H_2_ production in cyanobacterial mats were based on gas chromatographic analysis of intact mats (Skyring et al., 1988, 1989) and of gas bubbles carefully sampled from the surface of intact hypersaline cyanobacterial mats (Hoehler et al., 2001). The latter study, and more recently Burow et al. (2012) studying a coastal microbial mat also obtained a coarse depth distribution of H_2_ production by incubating 2 mm thick slices of mats from different depth horizons below the surface, showing maximal H_2_ production in the top 2 mm of the mat during night time. These findings were used to hypothesize that H_2_ production by ancient microbial mats and subsequent escape of the H_2_ to space was a major mechanism for facilitating the oxidation of the primitive Earth (Hoehler et al., 2001; Jørgensen, 2001). A series of elegant follow up studies combining biogeochemical process measurements with modern molecular tools, have (i) identified filamentous non-heterocystous cyanobacteria as the major H_2_ producers in such mats (Burow et al., 2012; Marschall et al., 2012), (ii) demonstrated cyanobacterial fermentation as the major H_2_ producing process (Burow et al., 2012; Lee et al., 2014), and (iii) demonstrated that sulfate reducing bacteria (SRB) are predominant hydrogenotrophs in cyanobacterial mats (Burow et al., 2014). There is thus increasing evidence that fermentative H_2_ and organic acid production is a key component in microbial mat biogeochemistry facilitating close interactions between cyanobacteria, anoxygenic phototrophs and heterotrophic bacteria (Otaki et al., 2012; Lee et al., 2014).

Despite an increasing interest in understanding the production and consumption of H_2_ in the environment, very few studies have described the fine scale distribution and dynamics of H_2_ (Witty, 1991; Ebert and Brune, 1997). Part of the reason has been lack of suitable technology (Hübert et al., 2011). Conventional Clark-type electrochemical H_2_ microsensors, which are based on the oxidation of H_2_ at a positively charged platinum electrode in an acidic KCl containing electrolyte (Wang et al., 1971; Witty, 1991), often suffer from unstable signals and calibration drift when used in natural systems. This has limited their applicability, especially in environments like sediments and microbial mats, where H_2_ measurements were hampered by sulfide interference on the measuring signal.

A sulfide-sensitive amperometric H_2_ microsensor based on the use of non-aqueous electrolyte is commercially available (Unisense A/S, Denmark), and a robust version of the sensor has proven useful for quantifying H_2_ production in vials with cyanobacterial cultures (Kothari et al., 2012, 2014). This sensor has very recently been employed for first microscale H_2_ measurement in intertidal microbial mats (Hoffmann et al., 2015) showing pronounced accumulation and eﬄux of H_2_ in darkness driven by cyanobacterial fermentation in the upper mm’s of the mat. The microenvironmental dynamics of H_2_ in hypersaline water covered mats remain to be studied in more detail, as these mat types often exhibit high sulfide levels causing interference on the commercial H_2_ microsensor The sensor design has now been further improved and a sulfide-insensitive H_2_ microsensor was recently developed (Nielsen et al., 2015). In the present study we use these microsensors for studying the H_2_ microenvironment and its relation to light, O_2_, pH and H_2_S micro gradients in coastal and hypersaline microbial mats. We demonstrate pronounced H_2_ dynamics during experimental light-dark shifts, and discuss the role of H_2_ for biogeochemical processes in microbial mats.

## Materials and Methods

### Experimental Setup

We studied H_2_ dynamics in two different microbial mats, both harboring a 1–3 mm thick dark-green surface layer with dense populations of filamentous non-heterocystous cyanobacteria, and anoxygenic *Chloroflexi*-like phototrophs.

#### Hypersaline Mat

Dense biofilms of filamentous cyanobacteria were retrieved from the top layer of a hypersaline microbial mat sampled in a salt evaporation pond of Saline de Giraud, Camargue, France. The mat locality and a detailed description of the biogeochemistry and microbial composition of the mat are presented elsewhere (Fourcans et al., 2004; Wieland et al., 2005). Microbial mat samples were transported to our laboratory and were kept in trays with aerated brine at *in situ* salinity (~80–100 ppt) under a 12 h light-12 h dark period in a thermostated room at 16°C. The mat was covered by a 2-3 mm thick deep-green biofilm of motile filamentous cyanobacteria. Microscopic investigations of the biofilm showed a dominance of morphotypes similar to *M. chtonoplastes* mixed with other motile filaments of *Oscillatoria* sp. and *Spirulina* sp. The upper millimeters of the mat remained non-sulfidic due to a conspicuous layer of oxidized iron below the cyanobacterial layer that buffered against sulfide formation in the uppermost mat layer during night-time (see details in Wieland et al., 2005).

Prior to experiments, a small piece of the surface biofilm was transferred to a small 3–4 mm high and 8 mm wide glass beaker with a thin layer of semisolid agar at ~38–40°C. During subsequent cooling the biofilm bottom and side adhered to the solidifying agar leaving the upper biofilm surface uncovered. The small beaker was mounted in a flow chamber (Lorenzen et al., 1995) with the biofilm surface flush with a larger agar slab, and aerated brine (90 ppt, 25°C, pH 8) was constantly circulated over the biofilm surface. The air-saturated brine contained 154 μmol O_2_ l^-1^ according to a table compiled from published empirical solubility equations^1^. Illumination was provided with a fiber-optic halogen lamp equipped with a collimating lens (KL-2500, Schott, Germany), where the irradiance was regulated with a built-in neutral density screen. During long-term incubations the lamp was turned on and off at defined times by an electrical switch with a timer. Irradiance levels at defined lamp settings were determined with a quantum irradiance meter (LI-250, LiCor, USA) equipped with a small spherical irradiance sensor (Walz GmbH, Germany).

#### Coastal Mat

Coastal microbial mat samples were collected in small acrylate coring tubes from the upper air-exposed yet moist part of a sandbar in Limfjorden near Aggersund, Denmark (57°00′02.15N; 9°17′12.89E). The mat undergoes irregular cycles of inundation and air exposure depending on prevailing wind directions and consisted of well sorted fine grained sand bound together by a dense 2–3 mm top layer of motile filamentous cyanobacteria (*M. chtonoplastes* and *Oscillatoria* sp.) and some green filamentous anoxygenic phototrophs *Chloroflexus* sp. and exopolymers on top of a black sulfidic layer, which also contained filamentous sulfide oxidizing bacteria (*Beggiatoa* sp.; Lassen et al., 1992). The mat samples were incubated for 1–2 days in aerated seawater under moderate illumination by halogen lamps (~100–200 μmol photons m^-2^ s^-1^) prior to experiments. During this time, the surface became densely covered by a dense layer of filamentous cyanobacteria (Supplementary Figure S1A). For comparison, we also sampled and investigated permanently submerged sediment samples from the same locality that were predominated by a dense benthic diatom film (Supplementary Figure S1B); these samples were obtained at the same location but from a sandier and less sulfidic sediment that was permanently water covered.

Experiments were conducted with the core samples mounted in an aquarium 1 cm below the surface of continuously aerated seawater (25 ppt, 21–22°C), which was circulated over the mat by a gentle airstream from a Pasteur pipette. The air-saturated seawater contained 240 μmol O_2_ l^-1^ according to a table compiled from published empirical solubility equations^1^. Illumination was provided by a halogen lamp bulb, where the irradiance was regulated by varying the distance to the mat surface. During long-term incubations, the lamp was turned on and off at defined times by an electrical switch with a timer. Irradiance levels at defined lamp distance were determined with a quantum irradiance meter (LI-250, LiCor, USA) equipped with a small spherical irradiance sensor (Walz GmbH, Germany).

Inhibition of sulfate reduction in the mat was done by incubating a mat sample in aerated and stirred sea water with 2.5 mM sodium molybdate for 6 h prior to measurements. Using published diffusion coefficients (D) of molybdate in water (9.91⋅10^-6^ cm^2^ s^-1^; Li and Gregory, 1974) and gel (6.48⋅10^-6^ cm^2^ s^-1^; Mason et al., 2005), we estimated the penetration depth of molybdate after *t* = 6 h of incubation as L = 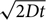 assuming a one-dimensional diffusion geometry (Berg, 1983). This showed that after 6 h of incubation, molybdate penetrated about 5.3–6.5 mm into the microbial mat ensuring sufficient exposure of SRB’s in the complete photic zone as well as several mm’s of the underlaying aphotic zone of the mat.

### Microsensor Measurements of O_2_, pH, H_2_S, and H_2_

Chemical microprofiles were measured with electrochemical microsensors for O_2_, H_2_S, pH and H_2_ with tip diameters of 10–70 μm (Unisense A/S, Denmark). Construction of electrochemical O_2_, pH, and H_2_S microsensors, their calibration and application have been described in previous publications (Revsbech and Jørgensen, 1986; Revsbech, 1989; Kühl et al., 1996, 1998; Kühl and Revsbech, 2001).

The H_2_ microsensor is constructed like a Clark-type O_2_ microsensor (Revsbech, 1989) and consists of an outer casing sealed by a thin silicon rubber membrane, and an internal measuring microanode polarized at +0.6 to +1.0 V relative to an internal reference electrode. The casing is filled with an organic electrolyte and this configuration facilitates a stable measurement of H_2_ via its oxidation at the measuring anode. The H_2_ microsensor is commercially available and further details on the sensor and its calibrations can be obtained from the manufacturer’s website^2^. We tested the interference of several compounds, which can pass the silicon membrane of the H_2_ microsensor and react at the measuring anode. Compounds like dimethyl sulfide (DMS) and methyl mercaptan can strongly affect the microsensor performance and seem to have a poisoning effect, but levels of these potential interfering agents in microbial mats have been found to be in the lower nM range (Visscher et al., 2003) and should thus not affect our measurements significantly. We found no interference from carbon monoxide, which has been shown to be present in hypersaline microbial mats during daytime (Hoehler et al., 2001) and is a known interfering agent of amperometric H_2_ sensors with aquatic electrolytes (Hübert et al., 2011). No sensitivity to light was observed. In the absence of a H_2_S shield, dissolved H_2_S is a major interfering substance and for a given concentration gives rise to a signal of ~20–30% of the signal measured for the same concentration of H_2_ (Nielsen et al., 2015). However, by mounting a thin outer capillary containing ZnCl_2_ in propylene carbonate and sealed with a thin silicone membrane, sulfide insensitive H_2_ microsensors can be constructed (Nielsen et al., 2015). We did not repeat tests of CO, DMS, and methyl mercaptan interference on the sulfide insensitive H_2_ sensors, but such interference will not be larger than on the unshielded sensors.

The new H_2_S insensitive H_2_ microsensor (see Nielsen et al., 2015 for details on construction and sensor design) exhibits a linear response from 0 to 100% H_2_. By varying the thickness and diameter of the silicone membrane sealing the microsensor tip as well as the distance from the membrane to the internal measuring anode, we could manufacture H_2_ microsensors with various measuring characteristics. Sensors without a H_2_S shield could be constructed with a very fast response time of <0.2 s, but they also exhibited a relatively large stirring sensitivity, which can cause severe measuring artifacts, especially when measuring concentration gradients within a gradient of flow, e.g., in the diffusive boundary layer above sediments and biofilms (Revsbech, 1989; Klimant et al., 1995). Hydrogen gas microsensors with a lower stirring sensitivity can be constructed by using smaller silicone membrane diameters and a longer internal diffusion path, and here the presence of a H_2_S shield contributes to the latter. The sulfide insensitive H_2_ microsensors used in this study exhibited a sensitivity of 1.5–5 pA μM^-1^ H_2_, with a negligible stirring sensitivity and a 90% response time of ~20–40 s. In all cases, the new H_2_ microsensors exhibited low and stable zero currents (1–10 pA) and a temperature sensitivity similar to other amperometric microsensors, i.e., an increase in sensor signal of 2–3% per °C.

We calibrated the sensor in salt water flushed with various defined amounts of H_2_, either by help of a gas-mixing unit or by using commercially available defined mixtures of H_2_ and N_2_. Hydrogen data were either expressed as partial pressures (%H_2_ saturation) or in molar concentration units. The H_2_ concentration in saturated water at experimental salinity and temperature was calculated from tabulated values of H_2_ solubility according to Wiesenburg and Guinasso (1979).

The amperometric microsensors were used in connection with a pA-meter (PA2000 and Microsensor Multimeter, Unisense A/S, Denmark), while the pH microsensors were used with a standard calomel reference electrode both connected to a high impedance mV meter (Microsensor Multimeter, Unisense A/S, Denmark). Measuring signals were either recorded on a stripchart recorder (BD-25, Kipp&Zonen, Netherlands) or via an A/D converter (Unisense A/S, Denmark) connected to a PC. Microsensors were mounted in a motorized micromanipulator that was mounted on a heavy stand and was remotely controlled by a PC-interfaced motor controller (Unisense A/S, Denmark). Automated data acquisition and positioning of microsensors was done with commercial software (*Profix* and *Sensor TracePro*, Unisense A/S, Denmark). The microsensors were inserted into the biofilm vertically from above in defined steps of 100–200 μm.

The eﬄux of H_2_ from the microbial mats, J(H_2_) quantified net H_2_ production and was calculated from measured concentration gradients using Fick’s firs law, J(H_2_) = D ^∗^ (dC/dz), where D is the molecular diffusion coefficient of H_2_ at experimental temperature and salinity and dC/dz is the linear H_2_ concentration gradient in the diffusive boundary layer above the mat. Similar flux calculations, J(O_2_) were done with O_2_ concentration profiles to quantify net photosynthesis in the light and dark O_2_ uptake rates, using the molecular diffusion coefficient of O_2_ at experimental temperature and salinity. Diffusion coefficients were taken from Broecker and Peng (1974) and corrected for temperature and salinity according to Li and Gregory (1974): D(O_2_) = 2.05⋅10^-5^ cm^2^ s^-1^ and D(H_2_) = 3.93⋅10^-5^ cm^2^ s^-1^ at 21°C and 25 ppt; D(O_2_) = 2.04⋅10^-5^ cm^2^ s^-1^ and D(H_2_) = 3.90⋅10^-5^ cm^2^ s^-1^ at 25°C and 90 ppt.

### Microsensor Measurements of Scalar Irradiance

Light penetration in the coastal microbial mat was measured with a scalar irradiance microsensor (Lassen et al., 1992; Kühl et al., 1997; Kühl, 2005) connected to a sensitive fiber-optic spectrometer (QE65000, Ocean Optics, USA) that was interfaced to a PC running dedicated spectral acquisition software (Spectrasuite, Ocean Optics, USA). Mat samples were illuminated vertically from above with a fiber-optic halogen lamp equipped with a collimating lens (KL-2500, Schott, Germany), where the downwelling photon irradiance was regulated with a built-in neutral density screen to 500 μmol photons m^-2^ s^-1^. A scalar irradiance microsensor was mounted in a manually operated micromanipulator (MM33, Märtzhäuser GmbH, Germany) and inserted into the mat at a 45° angle relative to the vertically incident light. Measurements were corrected for the measuring angle, and depths are given as vertical depth below the mat surface. Data were normalized to the incident downwelling irradiance as measured with the scalar irradiance microsensor positioned in the light path at similar distance as the mat surface but over a black light absorbing well.

## Results and Discussion

We measured H_2_ dynamics in two different cyanobacterial mats: (i) a hypersaline mat with a pronounced layer of oxidized iron buffering the cyanobacterial top layer against sulfide exposure (Wieland et al., 2005), and (ii) a highly sulfidic coastal cyanobacterial mat (Lassen et al., 1992). Data from the hypersaline mat were measured with H*_2_* microsensors without a sulfide trap and in the absence of H_2_S, as checked with a H_2_S microsensor (data not shown). Data in the highly sulfidic coastal mat were measured with H_2_ microsensors equipped with a chemical sulfide trap in front of the measuring tip (Nielsen et al., 2015).

### Hydrogen Production in the Hypersaline Cyanobacterial Mat

**FIGURE 1:**
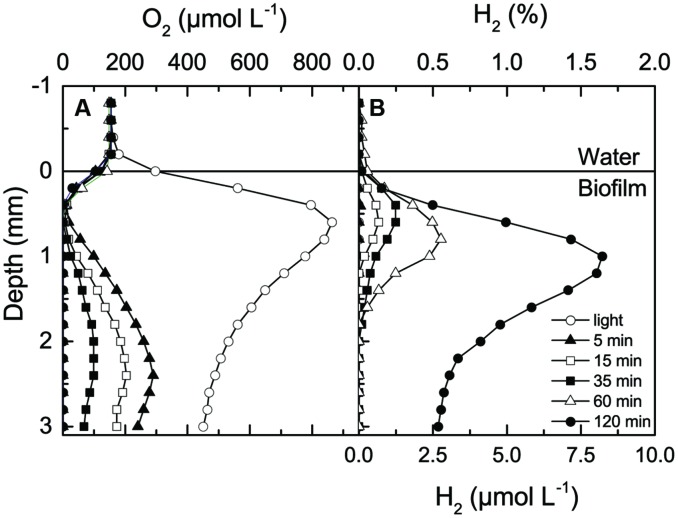
**Concentration profiles of O_2_**(A)** and H_2_**(B)** in a hypersaline microbial mat measured after 2.5 h under a photon irradiance of 800 μmol photons m^-2^ s^-1^ and as a function of time after darkening**.

When incubated under an irradiance of 800 μmol photons m^-2^ s^-1^ for 2.5 h, intense photosynthesis in the dense 2–3 mm thick hypersaline cyanobacterial biofilm lead to hyperoxic conditions reaching 4–5 times air saturation in the upper mm and supersaturating O_2_ levels throughout the whole sample, which was contained in a small glass container (**Figure 1A**). Upon darkening, O_2_ was most rapidly depleted in the region showing highest O_2_ production activity in light, and H_2_ was first detected in this zone after 15 min. As O_2_ became further depleted, H_2_ accumulated to higher concentrations and over a wider zone in the biofilm reaching a maximum of 8 μmol H_2_ L^-1^ (~1.6% H_2_) at 1 mm depth after 2 h in the dark. Hydrogen was consumed in the lowermost parts of the biofilm sample, which was constrained by the bottom of the small glass incubation container. The apparent migration of the H_2_ peak into slightly deeper layers probably reflects a shift in the relative balance between H_2_ production, consumption and transport, especially as the mat sample was confined in a small glass vial presenting a diffusion barrier ~4 mm below the mat surface.

**FIGURE 2:**
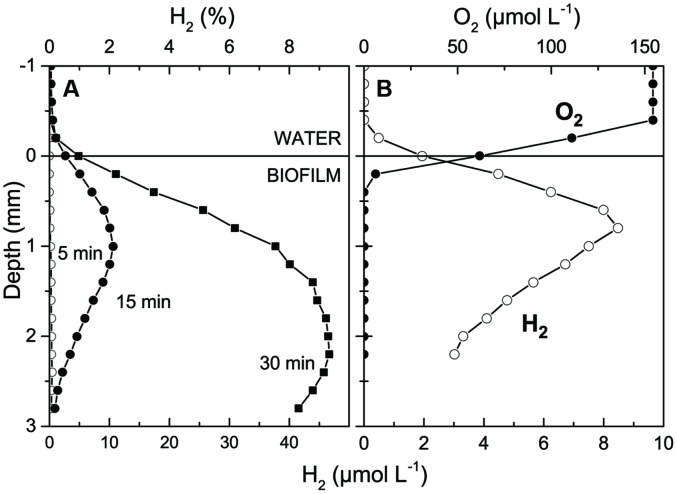
**(A)** Concentration profiles of H_2_ in a hypersaline microbial mat as a function of time after darkening. **(B)** Concentration profiles of H_2_ and O_2_ measured after 15 min darkness. The mat sample was incubated 2.5 h under a photon irradiance of 1800 μmol photons m^-2^ s^-1^ prior to darkening.

We note that the absolute amount of H_2_ produced in the mats after darkening apparently depended on the irradiance level during the previous light incubation. When we increased the irradiance to 1800 μmol photons m^-2^ s^-1^ for 2.5 h we again saw a very strong O_2_ accumulation in the sample but observed a much higher H_2_ production reaching ~2% H_2_ 15 min after onset of darkness. Maximal levels of 40–50 μM (8–9% H_2_) were reached in the upper millimeters of the mat within 30 min after darkening (**Figure 2A**). These H_2_ levels are higher than most other findings in more permanently submerged hypersaline mats that generally exhibit lower H_2_ accumulation than intertidal mats (**Table 1**).

**Table 1:** Comparison of maximal concentrations and fluxes of H_2_ reported in microbial mats. Listed in chronological order.

Sample origin and mat type	Salinity (ppt)	Maximal H_2_ concentration (μmol L^-1^)		Maximal H_2_ production (nmol H_2_ cm^-2^ h^-1^)		Source
		-molybdate	+molybdate	-molybdate	+ molybdate	
Hamelin Pool, Australia						Skyring et al. (1989), estimated from Figure 1
*Microcoleus* mat (smooth)	50–70	–	–	3-	17.9	
*Lyngbya* mat (tufted)	50–70	–	–	<0.1	0.58	
*Entophysalis* mat (pustular)	50–70	–	–	5.8	4.8	
Spencer Gulf, Australia						Skyring et al. (1989), Table 1
*Microcoleus* mat	45	–	–	0.125	36.3	
Guerrero Negro, Mexico						Hoehler et al. (2001), Figures 1 and 2, Table 1
*Lyngbya* mat (intertidal)	95	50	–	62.9	–	
*Microcoleus* mat (subtidal)	95	<0.5	–	0.35	–	
Elkhorn Slough, USA						Burow et al. (2012), estimated from Supplementary Figure S1
*Microcoleus* mat	35	–	–	49.5	–	
Guerrero Negro, Mexico						Hoffmann et al. (2015), estimated from Figures 3 and 4B
*Leptolyngbya* / *Microcoleus* mat (low tide)	40	~5	~20	11	–	
*Microcoleus* mat (mid tide)	40	~15	–	6.8	–	
*Microcoleus* / *Calothrix* mat (high tide)	40	~20	–	0.3	–	
Aggersund, Denmark						This study
*Microcoleus* mat	25	25–40	70	16.3	86.5	
Saline des Giraud, France						This study
*Microcoleus* mat	90	47	–	23.8	–	

We speculate that the apparent enhancement in H_2_ production with irradiance reflects a higher accumulation of storage products in the cyanobacteria enhancing subsequent dark fermentation. A similar explanation was proposed by Hoffmann et al. (2015), who measured sustained H_2_ production during darkness in intertidal microbial mats kept in a greenhouse for 1.5 years, and with an apparent positive correlation between the solar radiative flux during daytime and the night-time H_2_ production in the mats. A rigorous test of this hypothesis would require measurements of cyanobacterial photosynthate accumulation as well as H_2_ and fermentation products as a function of light incubation time and may be complicated by the cross-feeding of cyanobacterial fermentation products to other mat members such as SRB and Chloroflexi (Burow et al., 2014; Lee et al., 2014). Measurements on cyanobacterial cultures using methodology described by Kothari et al. (2014) may thus be more straightforward.

Alignment of H_2_ and O_2_ microsensor measurements showed that H_2_ diffused out of the mat in the dark (**Figure 2B**) with an estimated maximal H_2_ eﬄux of ~23.8 nmol H_2_ cm^-2^ h^-1^ amounting to about 13% of the diffusive O_2_ uptake of 182.2 nmol O_2_ cm^-2^ h^-1^ in the dark and 2% of the net photosynthetic O_2_ production in light of 1420 nmol O_2_ cm^-2^ h^-1^. Hoffmann et al. (2015) did similar measurements in different intertidal mats and found that H_2_ production in the dark amounted to 0.2–5% of net photosynthesis and 0.4–28% of O_2_ respiration.

**FIGURE 3:**
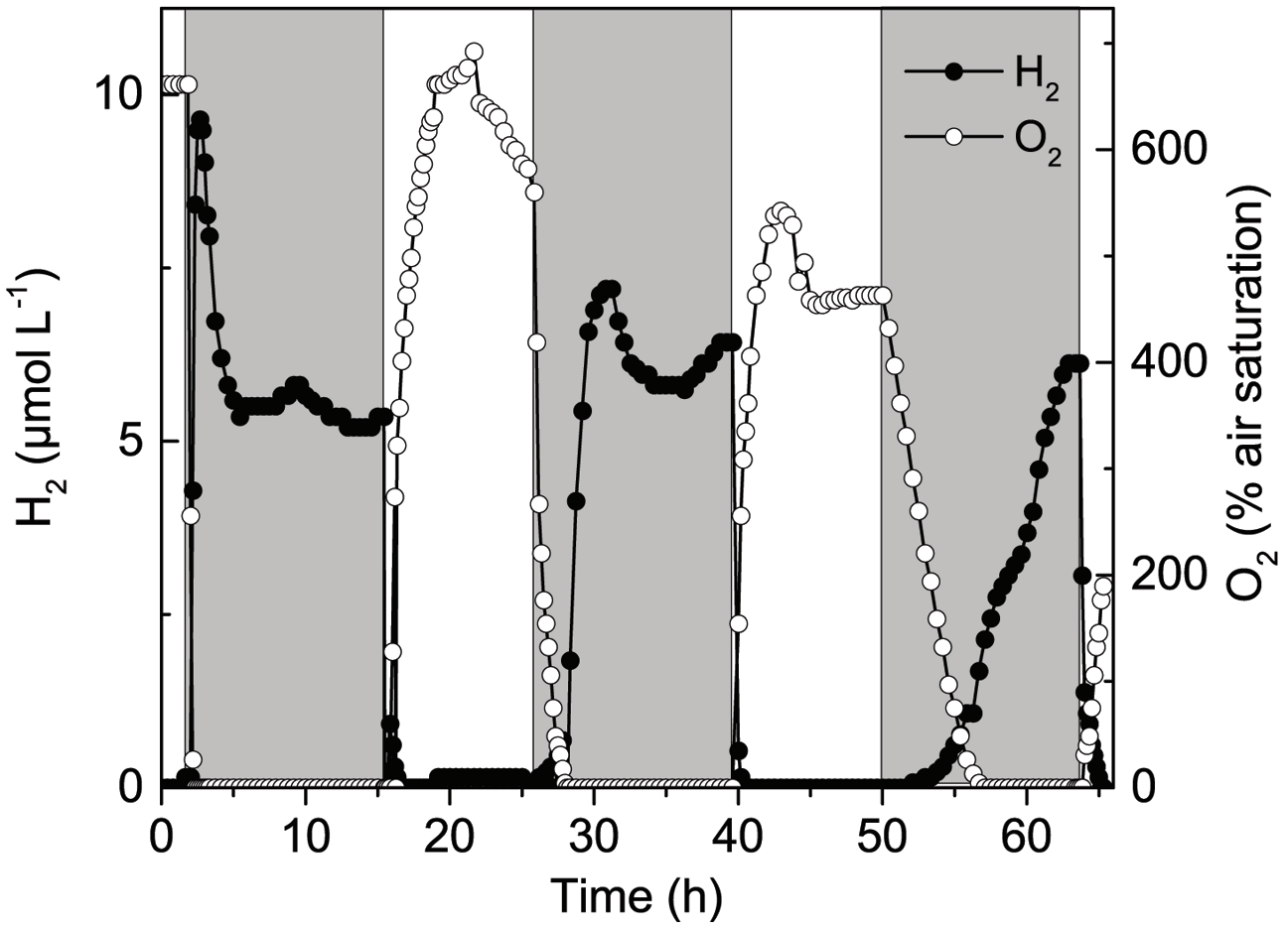
**Long-term measurements of H_2_ and O_2_ concentration at 0.8 mm depth in a hypersaline cyanobacterial mat sample incubated under a 14 h light (800 μmol photons m^-2^ s^-1^) and 10 h dark cycle**.

Long-term experiments with simultaneous O_2_ and H_2_ measurements over several days (14 h dark: 10 h light) with the microsensor tips positioned 0.8 mm below the mat surface, i.e., in the zone of maximal O_2_ production in the light, showed recurrent H_2_ production that persisted in the anoxic mat throughout the 14 h dark incubation period (**Figure 3**). Quantification of H_2_ production in *Lyngbya aestuarii* and *M. chtonoplastes* cultures showed similar long term persistence for >24 h (Kothari et al., 2014). The build-up of H_2_ was highest immediately after darkening and then leveled off after 1–2 h. Strong H_2_ depletion occurred rapidly after onset of the illumination leading to O_2_ accumulation from photosynthesis. Such depletion can be explained by several mechanisms such as (i) O_2_ inhibition of H_2_ production coupled with diffusive losses, (ii) H_2_ consumption with O_2,_ by e.g., Knallgas bacteria, and/or intermittent anoxygenic photosynthesis. However, our limited experimental data do not allow us to discriminate between the relative importance of these H_2_ consuming processes.

In the second light period, the apparently constant O_2_ level at this particular depth over many hours indicated formation of a gas bubble in the mat that acted as an O_2_ and H_2_ reservoir slowing the O_2_ depletion and the build-up of H_2_ after the subsequent light-dark shift. However, the slower O_2_ and H_2_ dynamic in subsequent light–dark shifts could also indicate an increasing substrate limitation as the light period may not have been sufficient to restock cyanobacterial storage products.

Overall, the observed H_2_ dynamics in the hypersaline mat is very similar to patterns recently observed in cyanobacterial cultures (Kothari et al., 2014) and intertidal microbial mats (Hoffmann et al., 2015). In comparison to other studies of H_2_ in hypersaline mats (**Table 1**), we found much higher H_2_ levels after darkening. The reasons for such high H_2_ accumulation remain to be studied in detail, but we speculate that the high content of oxidized iron in the upper layers of the Saline des Giraud mat (Wieland et al., 2005) may lead to less sulfate reduction and thus less consumption of H_2_, in contrast to most other hypersaline mat systems that often become highly sulfidic in darkness due to intense sulfate reduction in the top layers. However, the higher H_2_ levels may also simply reflect that the small glass incubation vial confined the sample to only a 3–4 mm thick top layer and thus did not allow a diffusive exchange and consumption in deeper more sulfidic mat layers. There is thus clearly a need for more detailed H_2_ and H_2_S measurements on deeper mat cores from Saline des Giraud.

### Hydrogen Dynamics in Coastal Cyanobacterial Mats

**FIGURE 4:**
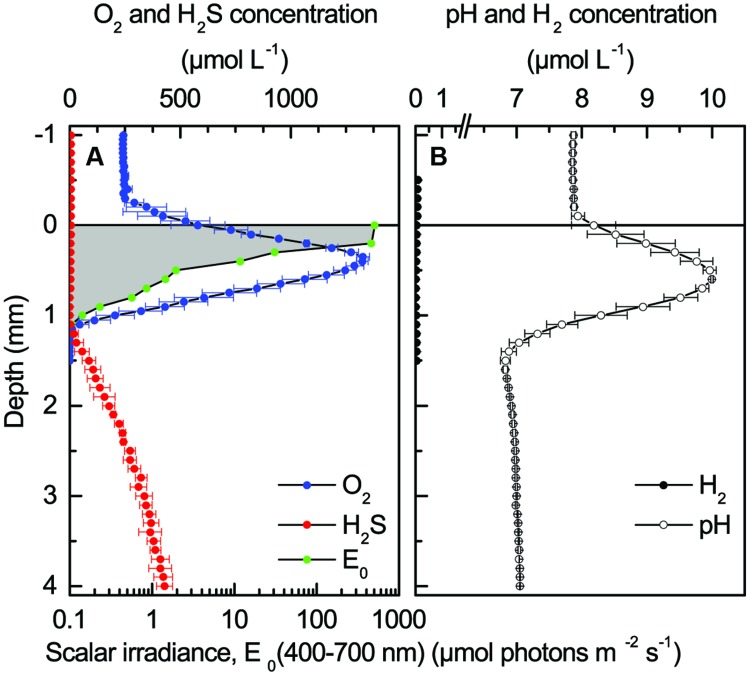
**Light and chemical gradients in a coastal cyanobacterial mat under an incident photon irradiance of 500 μmol photons m^-2^ s^-1^. (A)** Depth profiles of photon scalar irradiance, O_2_ and H_2_S concentrations. **(B)** Depth profiles of pH and H_2_ concentration. Symbols with error bars for chemical parameters represent the mean ± SD (*n* = 3).

More detailed microenvironmental analyses of H_2_ dynamics were done in a sulfidic coastal cyanobacterial mat (Supplementary Figure S1A), whereas measurements in coastal sediment with a surface biofilm of diatoms (Supplementary Figure S1B) showed no accumulation of H_2_ (data not shown). Spectral scalar irradiance measurements showed strong light attenuation with depth in the dense 1–2 mm thick top layer of the coastal mat, where distinct throughs in the transmission spectra indicated a high density of cyanobacteria with Chl *a* and phycobilins, as well as anoxygenic phototrophs with Bchl *a* and Bchl *c* (Supplementary Figure S2), with the latter being indicative of the presence of Chloroflexi. The euphotic zone for oxygenic photosynthesis was limited to the uppermost mm of the mat, wherein visible light (PAR, 400–700 nm) was attenuated to ~0.1 μmol photons m^-2^ s^-1^ (**Figure 4A**). Similar optical characteristics were found in samples from the same site by Lassen et al. (1992) >20 years ago.

#### Chemical Microenvironment in Light

The chemical conditions in the mat exhibited steep concentration gradients (**Figure 4**). Under high irradiance of PAR (500 μmol photons m^-2^ s^-1^), intense photosynthesis led to peak O_2_ concentrations of ~4.5 times air-saturation 0.5 mm below the mat surface. However, O_2_ only penetrated to ~1.2 mm in the light due to intense respiration and re-oxidation of reduced chemical species. Sulfide was produced by SRB in deeper mat layers, where H_2_S levels reached about 0.5 mM at 3 mm depth. Sulfide was re-oxidized by O_2_ in a thin zone around 1.2-1.4 mm depth. Strong photosynthesis caused a strong pH increase in the photic zone peaking at pH 10 around 0.5–0.7 mm below the mat surface, i.e., >2 pH units above the overlaying water pH of 7.9. With increasing depth, pH dropped by ~3 units reaching pH <7 in the sulfide oxidation zone before stabilizing around pH 7 in deeper mat layers. No H_2_ was detected in the upper millimeters of the illuminated mat. Measurements in another mat sample from the same habitat showed the same chemical zonations and extremes, albeit with a somewhat deeper O_2_ penetration depth in light of ~1.5 mm and a more heterogeneous distribution of H_2_S in deeper mat layers (Supplementary Figure S3).

#### Hydrogen Gas Production and Chemical Dynamics after Darkening

**FIGURE 5:**
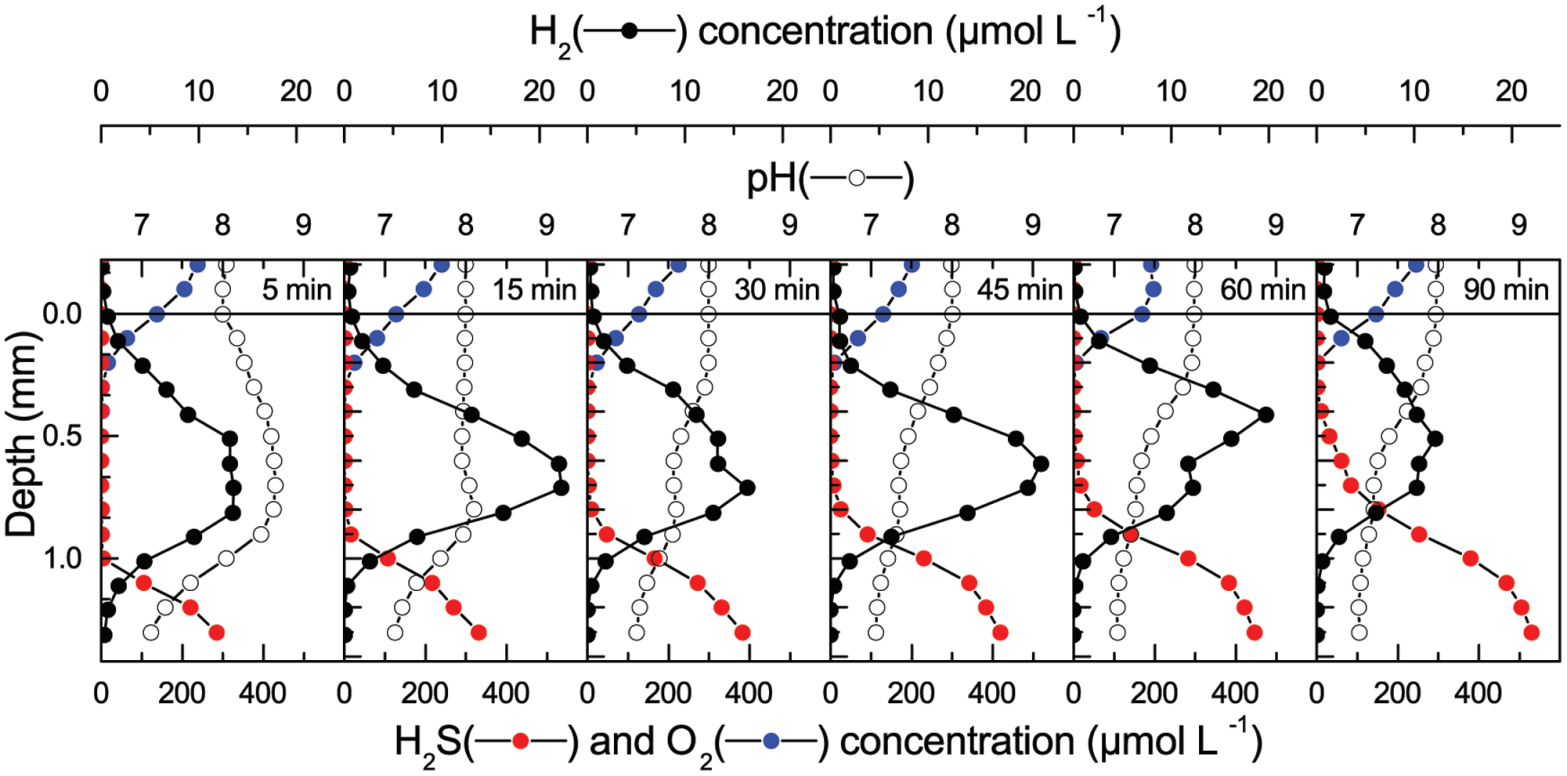
**Chemical gradients in a coastal cyanobacterial mat (same sample as in **Figure 4**) as a function of time after darkening.** Prior to darkening, the mat was incubated in aerated seawater under a photon irradiance of 500 μmol photons m^-2^ s^-1^.

**FIGURE 6:**
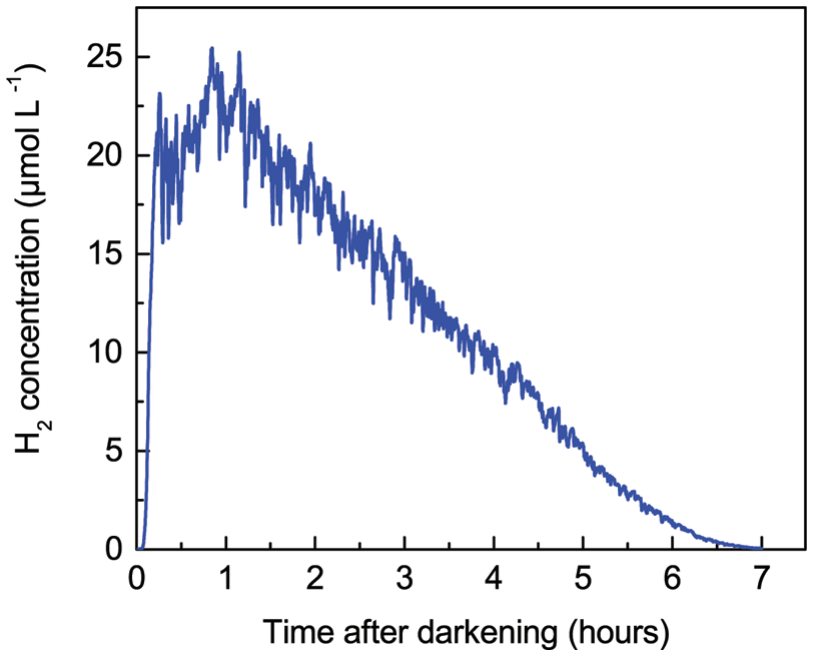
**Accumulation and depletion of H_2_ in a coastal cyanobacterial mat (same sample as in **Figure 4**) as a function of time after darkening.** Measurements were performed 0.6 mm below the mat surface in the zone of maximal H_2_ production (see **Figure 5**).

The chemical conditions in the coastal mat changed dramatically after darkening (**Figure 5**). Within 5 min after darkening, O_2_ became strongly depleted and only penetrated ~0.3 mm into the mat, with a further decrease in the O_2_ penetration depth to 0.2 mm over the following 85 min. The O_2_ and H_2_S concentration profiles were initially separated by a ~0.7 mm wide zone, wherein H_2_ accumulated rapidly after darkening. Peak concentrations were measured 0.5–0.7 mm below the mat surface increasing from ~13 μM H_2_ after 5 min to ~22 μM H_2_ after 45 min dark incubation. Over this time interval, pH in the H_2_ production zone decreased to a stable value of pH 7.2–7.8. Produced H_2_ diffused both toward the mat surface and toward the sulfidic zone, where it was consumed. After 45 min, H_2_ levels in the mat started to decrease, while sulfide levels continued to increase in the upper mat layers. Sulfide started to overlap with the O_2_ concentration profile after 90 min. Hydrogen levels in the mat continued to decrease slowly and in a second experiment complete H_2_ depletion was only found after about 7 h dark incubation as seen in **Figure 6**, where data from continuous measurements at 0.6 mm depth are shown. The observed pattern of rapid build-up followed by a slow decline in H_2_ concentration follows a similar pattern observed in studies of H_2_ evolution in cyanobacterial isolates (Kothari et al., 2012).

#### Hydrogen Accumulation in the Presence of Molybdate

**FIGURE 7:**
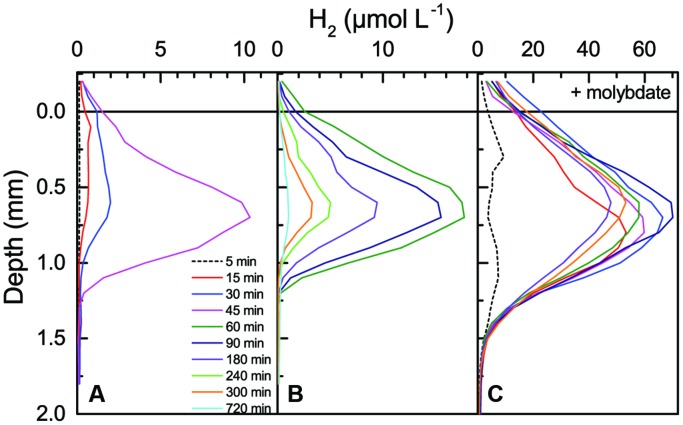
**Accumulation and depletion of H_2_ in a coastal cyanobacterial mat as a function of time after darkening (see numbers and color legends in the graph).** Prior to darkening, the mat was incubated under a photon irradiance of 500 μmol photons m^-2^ s^-1^ in aerated seawater for 8 h without **(A,B)** and for 6 h with 2.5 mM molybdate **(C)**. Note the use of different concentration ranges in the different panels.

Additional measurements of H_2_ production after onset of darkness were done in another coastal mat sample from the same habitat (**Figure 7**) that was incubated 8 h under a photon irradiance of 500 μmol photons m^-2^ s^-1^ prior to darkening. When incubated in normal seawater, the mat reached maximal concentrations of ~18 μM H_2_ at 0.5–0.7 mm depth within 60 min after darkening, where after H_2_ levels in the mat declined gradually to ~1.5 μM H_2_ after 720 min in darkness (**Figures 7A,B**). Thereafter, the same mat was incubated 6 h under a photon irradiance of 500 μmol photons m^-2^ s^-1^ in seawater with 2.5 mM molybdate, an inhibitor of sulfate reduction. Measurements in light at the end of this incubation showed a similar O_2_ and pH distribution, whereas H_2_S levels in the mat were much lower below the photic zone than in the absence of molybdate (Supplementary Figures S3 and S5); interestingly, a slight accumulation of H_2_ (reaching 1–1.5 μM) was also observed in deeper mat layers around 3 mm depth after the molybdate treatment, i.e., in the aphotic zone that exhibited high H_2_S levels in the absence of molybdate.

In presence of molybdate, the H_2_ accumulation in the mat after darkening was much stronger and H_2_ penetrated deeper into the mat (**Figure 7C**). Within 30 min after darkening, H_2_ concentrations reached peak values of >60 μM H_2_ 0.6–0.8 mm below the mat surface. The H_2_ concentrations remained high in the dark incubated mat for about 5 h and showed much slower H_2_ depletion than in the absence of molybdate. Without molybdate, the produced H_2_ penetrated to a depth of 1.1–1.3 mm where it became fully depleted in a relatively narrow zone (**Figures 7A,B**). In the presence of molybdate, H_2_ penetrated to a depth of almost 2 mm and the H_2_ concentration profile showed a much more gradual depletion with depth (**Figure 7B**). Both with and without molybdate, there was no indication of strong H_2_ depletion in the uppermost mat layer and the microprofiles showed an eﬄux of H_2_ into the overlaying water. The H_2_ eﬄux was strongly stimulated in the presence of molybdate (**Table 1**). In the absence of molybdate, the maximal H_2_ eﬄux 60 min after onset of darkness reached 16.3 nmol H_2_ cm^-2^ h^-1^ amounting to 5.5% of the dark O_2_ consumption (296.2 nmol O_2_ cm^-2^ h^-1^) and 1% of the net photosynthetic O_2_ production prior to darkening (1500 nmol O_2_ cm^-2^ h^-1^). In the presence of molybdate, the maximal H_2_ eﬄux reached 86.5 nmol H_2_ cm^-2^ h^-1^ amounting to 29% of the dark O_2_ consumption and 6% of the net photosynthesis.

Hoffmann et al. (2015) did a similar experiment in intertidal microbial mats showing stimulated H_2_ production reaching 4 times higher peak concentrations of H_2_ in the presence of molybdate reaching up to ~20 μM H_2_ in the upper mm of the mat (**Table 1**). However, their measurements with a sulfide sensitive H_2_ microsensor showed a H_2_ concentration peak on top of an apparent gradually increasing H_2_ concentration with depth, which can be interpreted as an sulfide interference on the microsensor signal (see below).

Bulk measurements of the H_2_ production of coastal and hypersaline mats (Skyring et al., 1989) and in the upper 2 mm of a coastal cyanobacterial mat (Burow et al., 2014) and two hypersaline cyanobacterial mats (Lee et al., 2014) all showed a strong stimulation of H_2_ production upon inhibition of sulfate reduction activity by addition of molybdate to the incubation vials. Molecular analyses of the microbial diversity and gene expression in such mats identified SRB as major hydrogenotrophs in the mat along with filamentous anoxygenic phototrophs belonging to the Chloroflexi (Burow et al., 2014; Lee et al., 2014). In the present study, we did not investigate the distribution and identity of SRB or Chloroflexi or their hydrogenase gene expression in the mat samples, but our H_2_ microsensor data strongly support the findings in other cyanobacterial mats identifying SRB as primary hydrogenotrophs in the upper mat layers.

A close spatial co-occurrence of SRB and cyanobacteria has been demonstrated in the upper millimeters of several microbial mat environments (e.g., Baumgartner et al., 2006; Fike et al., 2008), including observation of migratory behavior of motile SRB toward the photic zone (Krekeler et al., 1998). This has been ascribed to aerobic sulfate reduction (Canfield and Des Marais, 1991) and/or a possible aerotaxis combined with aggregation and high O_2_ respiration as a survival mechanism for SRB in the photic zone (Cypionka, 2000; Baumgartner et al., 2006). But the presence of SRB within the photic zone also reflects the easy access of SRB to both electron donor and acceptor immediately after onset of darkness and onset of fermentative H_2_ production (Lee et al., 2014), and we note that some SRB can also catalyze the oxidation of H_2_, organic substrates and inorganic sulfur species with O_2_ as an electron acceptor (Dannenberg et al., 1992). Metabolic flexibility including a versatile H_2_ metabolism and chemotaxis of SRB may thus enable them to thrive in the highly variable chemical microenvironment of the photic zone in microbial mats.

#### Photo-Stimulation of H_2_ Production

**FIGURE 8:**
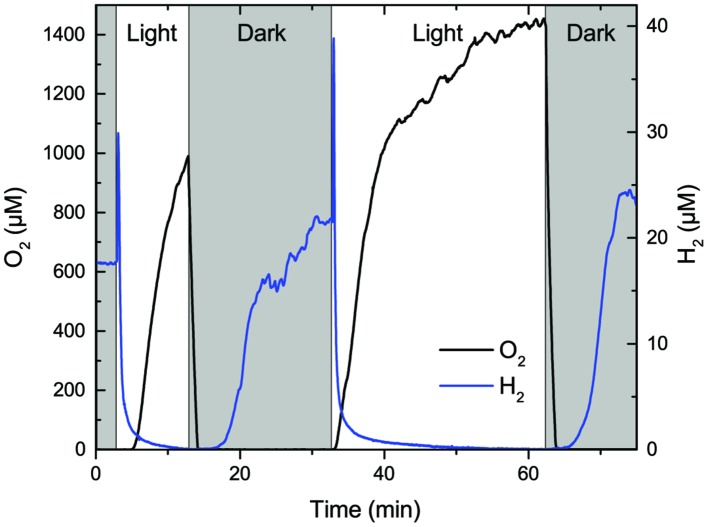
**Dynamics of H_2_ and O_2_ concentration measured 0.6 mm below the surface of a coastal cyanobacterial mat in darkness and upon onset of illumination (500 μmol photons m^-2^ s^-1^)**.

Simultaneous measurements of O_2_ and H_2_ concentration in the coastal mat at a depth of 0.6 mm, i.e., within the zone of maximal H_2_ production in the dark and photosynthetic O_2_ production in the light, showed pronounced dynamics (**Figure 8**). In the dark, O_2_ was depleted completely and H_2_ concentrations reached levels of 20–25 μM H_2_ after 15–20 min. However, immediately after onset of illumination we observed a burst in H_2_ production driving local concentrations up to 30–40 μM H_2_. This burst only lasted for 20–30 s, where after H_2_ became rapidly depleted as O_2_ from photosynthesis accumulated to super saturating concentration levels in the mat. Similar measurements (data not shown) in depth horizons closer to the mat surface showed a shorter and less intense burst due to more rapid O_2_ accumulation and thus faster H_2_ depletion, whereas measurement in deeper mat layers showed a less intense build-up of H_2_ upon onset of illumination due to strong light limitation; at 0.8 mm depth we observed no photo stimulation of H_2_ production. Such intermittent pulses of H_2_ upon illumination have been ascribed to direct biophotolysis in cyanobacteria involving a bidirectional Ni–Fe hydrogenase (Appel et al., 2000). While our data give first evidence that such biophotolysis can occur in the uppermost parts of cyanobacterial mats, the process is limited to <1 min after onset of illumination and thus plays a very minor role for the total H_2_ production.

#### Sulfide Interference on H_2_ Microsensor Measurements

Our measurements in the hypersaline mat were done under absence of sulfide (checked by H_2_S microsensor measurements) due to a pronounced layer of oxidized iron buffering against accumulation of free sulfide in the photic zone during darkness (Wieland et al., 2005). Under such conditions, the commercially available H_2_ microsensor from Unisense performs well and gives accurate quantifications of H_2_ concentrations. However, this sensor is also sensitive to hydrogen sulfide giving rise to about 20–30% of the signal for a given H_2_S concentration as compared to the same H_2_ concentration, and exposure to high H_2_S levels can also affect sensor calibration (cf. H_2_ microsensor manual available at: www.unisense.com/manuals/). Typical H_2_S concentrations in the upper millimeters of hypersaline and coastal mats can reach up to 500 μM just below the photic zone in light or in the uppermost mat layers in darkness (Wieland and Kühl, 2000). A strong gradient of increasing H_2_S concentration with depth would thus be detected in H_2_ microsensor measurements giving rise to false H_2_ signals of up to >50 μM and typically showing a continuously increasing H_2_ level with depth in the mat following the increasing H_2_S concentration. Hoffmann et al. (2015) measured with the sulfide-sensitive H_2_ microsensor in three types of intertidal mats. In the upper tidal apparently less sulfidic mat they found a clear peak of H_2_ production overlapping with the photic zone, while measurements in the other mats showed that such a H_2_ production peak was overlayed by a strong continuously increasing H_2_ concentration with depth (cf. Figure 3 in Hoffmann et al., 2015). While the authors did not report on H_2_S measurements in their mats, we suggest that their measurements in deeper mat layers may include H_2_S interference. We have observed similar patterns when using sulfide sensitive H_2_ microsensors in the coastal mat from Aggersund (data not shown). Such interference would also affect subsequent rate calculations on measured H_2_ concentration profiles, especially in deeper zones. With the new sulfide-insensitive microsensor it is now possible to measure in strongly sulfidic microbial mats without such potential artifacts.

## Conclusion

Our measurements demonstrated distinct microscale dynamics of H_2_ in hypersaline and coastal microbial mats that are densely populated by filamentous cyanobacteria. We found a pronounced build-up of H_2_ in the upper millimeters of such mats upon darkening, while more oxidized coastal sediments with a surface biofilm of diatoms did not show any H_2_ accumulation. In general, our results support other recent demonstrations of strong H_2_ production in mat-forming cyanobacteria (Kothari et al., 2012, 2014) and intact microbial mats (Hoehler et al., 2001; Burow et al., 2012; Hoffmann et al., 2015), where cyanobacterial fermentation of photosynthate in darkness is the major H_2_ source. Such H_2_ formation upon light–dark shifts in the upper photic zone thus seems inherent in many coastal and hypersaline microbial mats and presents an important, yet intermittent energy source that together with photosynthate fermentation products can fuel anaerobic respiration processes such as sulfate reduction (Lee et al., 2014).

In conclusion, this first application of a new H_2_ microsensor (Nielsen et al., 2015) in concert with microsensors for O_2_, pH, H_2_S, and scalar irradiance demonstrated a pronounced potential for H_2_ production in the photic zone of microbial mats. Strong intermittent H_2_ accumulation (up to 30–40 μM H_2_) and eﬄux of H_2_ to the overlaying water originated in the uppermost cyanobacterial layers, with the most intense H_2_ formation in the depth horizons exhibiting maximal photosynthesis in the light. In the dark, H_2_ first accumulates in the uppermost mm of the mat and is then released to the overlaying water and consumed over 6–7 h by anaerobic respiration. Depletion of H_2_ within the mat was strongly inhibited by molybdate addition pointing to SRB as major hydrogenotrophs in the mat. Strong H_2_ production by biophotolysis was observed in the uppermost anoxic mat layers (0.2–0.8 mm) immediately (for <1 min) after onset of illumination but was quickly inhibited by oxygenic photosynthesis and did not contribute significantly to the H_2_ production, which was primarily observed in the dark. The new microsensors allow detailed studies of H_2_ dynamics in sulfidic environments at high spatio-temporal resolution. Such studies have until recently been limited to mm-scale measurements of net H_2_ production from incubated mat samples using gas chromatography or other bulk phase measurements. Here we have focused on costal and hypersaline cyanobacterial mats, but the new H_2_ sensor is also suitable for measurements at higher temperatures (up to 60°C; Nielsen et al., 2015) and we are currently investigating H_2_ dynamics in hot spring microbial mats, where H_2_ metabolism often plays a major role (Spear et al., 2005) although the relative importance of H_2_ and H_2_S as an energy source remains debated (D’Imperio et al., 2008).

## Author Contributions

Planned and designed experiments (MN, NR, MK). Performed experiments and analyzed data (MN, NR, MK). Wrote the article (MK with editorial help by MN and NR).

## Conflict of Interest Statement

The authors declare that the research was conducted in the absence of any commercial or financial relationships that could be construed as a potential conflict of interest.

## Footnotes

1 http://www.unisense.com/files/PDF/Diverse/Seawater%20&%20Gases%20table.pdf

2 www.unisense.com
