# Supplementary material for: Microsensor measurements of hydrogen gas dynamics in cyanobacterial microbial mats
Source: Front Microbiol. 2015 Jul 21;6:726. doi: 10.3389/fmicb.2015.00726 (PMC4508582; doi:10.3389/fmicb.2015.00726)

Supplementary Material

Microsensor Measurements of Hydrogen Gas Dynamics in Cyanobacterial Microbial Mats

Michael Nielsen[1], Niels Peter Revsbech[2], Michael Kühl[2,3,*]

[1]Department of Bioscience – Microbiology, Aarhus University, Ny Munkegade 116, DK-8000 Aarhus C, Denmark
[2]Marine Biological Section, Department of Biology, University of Copenhagen, Strandpromenaden 5, DK-3000 Helsingør, Denmark
[3]Plant Functional Biology and Climate Change Cluster, University of Technology Sydney, Ultimo, NSW 2007, Australia

*** Correspondence:** Michael Kühl, Marine Biological Section, Department of Biology, University of Copenhagen, Strandpromenaden 5, Helsingør, DK-3000, Denmark.
mkuhl@bio.ku.dk

Supplementary Figures

Supplementary Figure 1. Photographs of a coastal microbial mat sample covered with a dense top layer of filamentous cyanobacteria (left panel), and a coastal sediment sample covered with a biofilm of pennate diatoms (right panel). The left panel also shows a H_2S and a pH microsensor positioned just above the microbial mat surface.

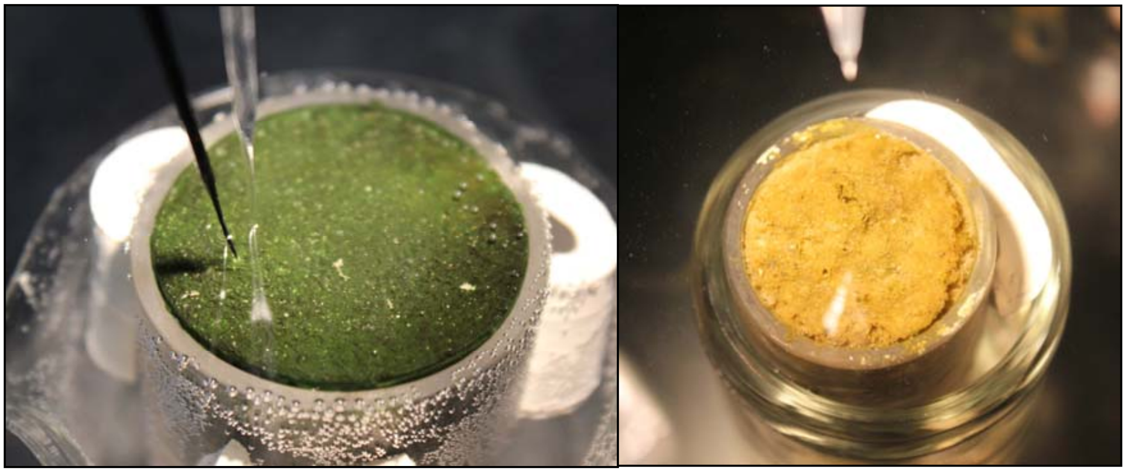

Supplementary Figure 2. Spectral scalar irradiance measurements (in % of incident downwelling irradiance) as a function of depth in 2 samples of the coastal microbial mat (A, B). Throughs in the transmission spectra correspond to major absorption maxima of the dominant photopigments (Chl a: 440 and 675 nm; Phycobiliproteins, PB: 575 and 620 nm, Bchl c: 750 nm; Bchl a: 800 and 860 nm) in the mat as indicated with dashed lines in the figure. Corresponding chemical micro profiles are shown in Fig. 4 and SFig. 3 for panel A and B, respectively.

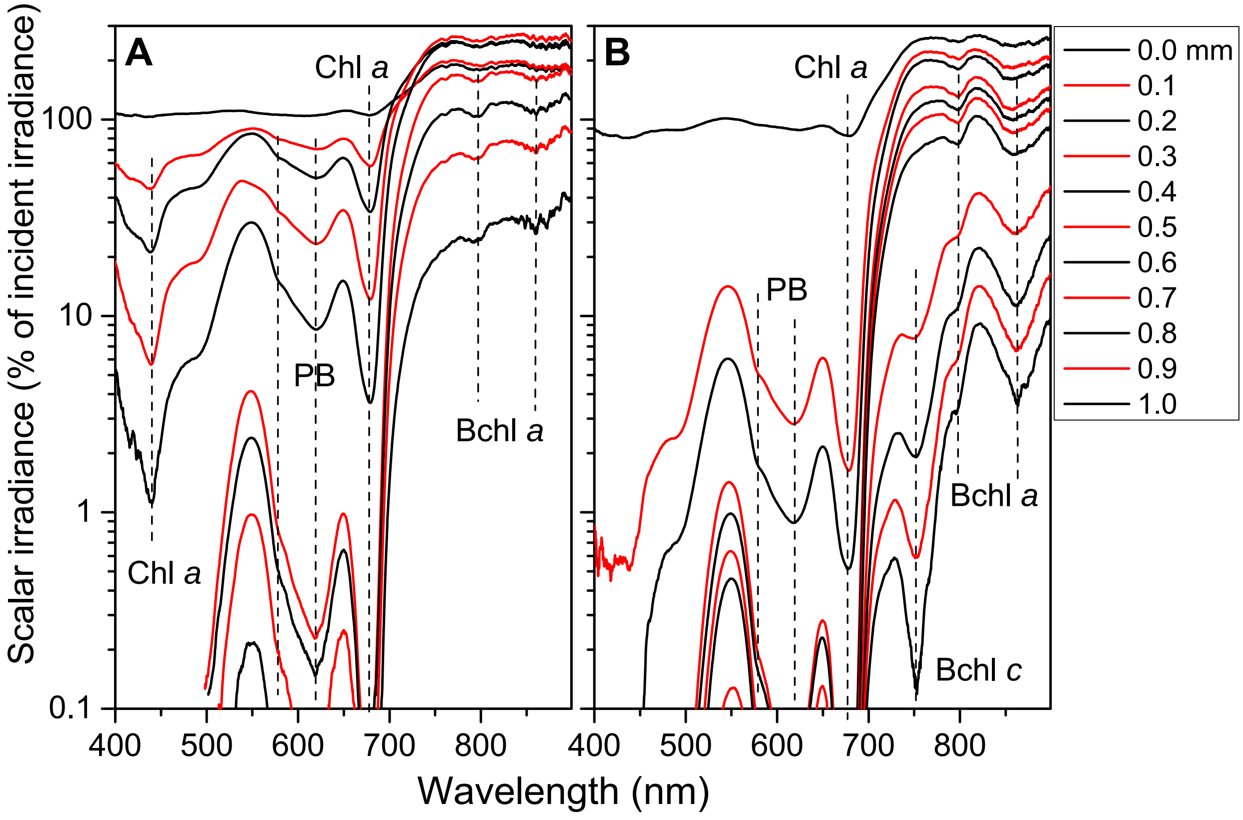

Supplementary Figure 3. Light and chemical gradients in a coastal mat (sampled from the same habitat as the sample shown in Fig. 4 and SFig. 1A) under an incident photon irradiance of 500 µmol photons m^{-2} s^{-1}. Left panel shows depth profiles of photon scalar irradiance, O_2 and H_2S concentrations. Right panel shows depth profiles of pH and H_2 concentration. Symbols with error bars represent the mean ± standard deviation (n=3).

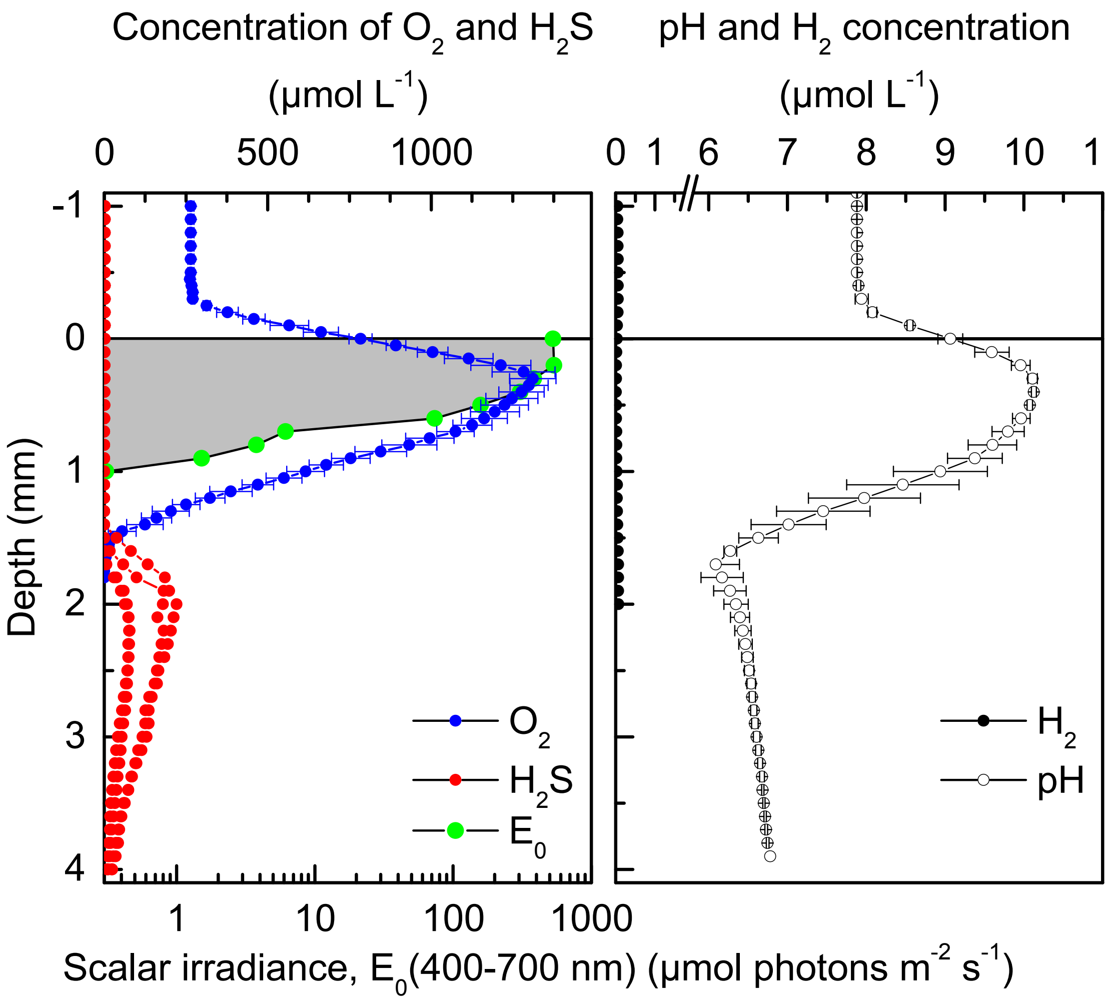

Supplementary Figure 4. Chemical gradients in a coastal mat (same sample as in SFig. 3) in darkness. Left panel shows depth profiles of O_2 and H_2S concentration (n=3). Right panel shows depth profiles of pH (n=3) and H_2 concentration (n=1). The H_2 microprofile represents the maximal H_2 concentrations measured 60 minutes after onset of darkness (the full dynamics of H_2 concentration profiles as a function of time after darkening is given in Fig. 7A and B).

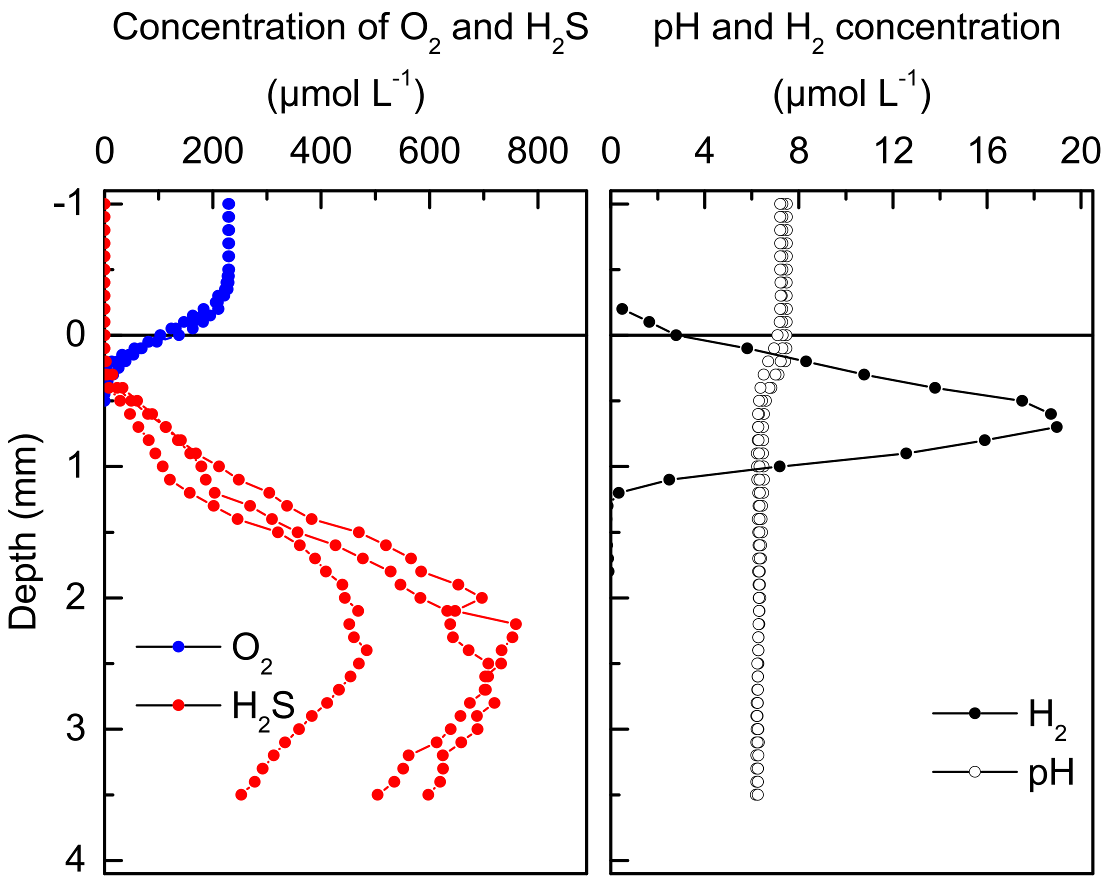

Supplementary Figure 5. Light and chemical gradients in a coastal mat (same sample as in SFig. 3 and SFig. 4) under an incident photon irradiance of 500 µmol photons m^{-2} s^{-1} after incubation in seawater with 2.5 mM molybdate. Left panel shows depth profiles of O_2 and H_2S concentrations. Right panel shows depth profiles of pH and H_2 concentration.

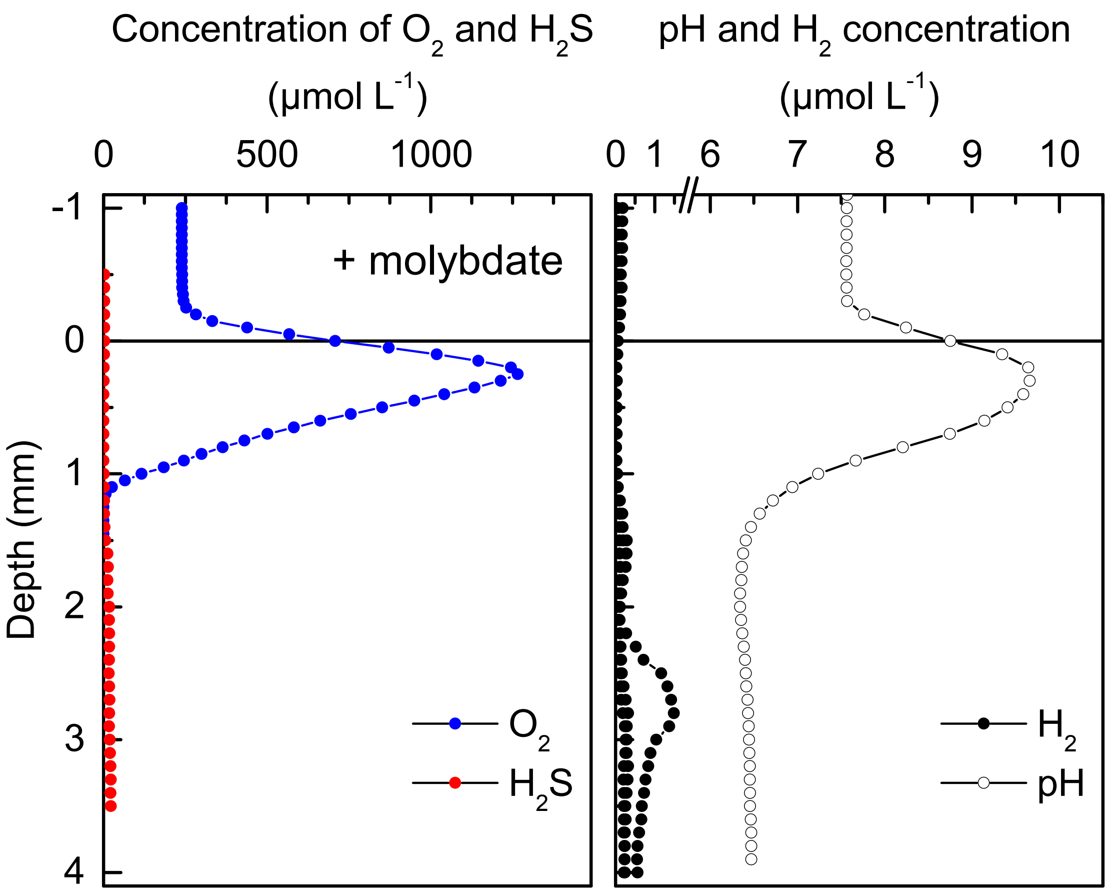

Supplement: Supplementary file 1 [file Presentation_1.PDF]
